# Implementing a Chronic Disease Self-Management Program into China: The Happy Life Club™

**DOI:** 10.3389/fpubh.2014.00181

**Published:** 2015-04-27

**Authors:** Colette Joy Browning, Hui Yang, Tuohong Zhang, Anna Chapman, Shuo Liu, Joanne Enticott, Shane Andrew Thomas

**Affiliations:** ^1^Royal District Nursing Service Institute, St Kilda, VIC, Australia; ^2^School of Primary Health Care, Monash University School of Primary Health Care, Monash University, Melbourne, VIC, Australia; ^3^Research Center for Ageing and Health Services, Department of Global Health, Peking University, Beijing, China; ^4^Department of Social Medicine and Management, School of Public Health, Peking University, Beijing, China; ^5^The University of Adelaide, Adelaide, SA, Australia

**Keywords:** chronic disease self-management, motivational interviewing, diabetes, older people, China

## Abstract

China is experiencing population aging, increased prevalence of chronic diseases, and reductions in the frequency of healthy lifestyle behaviors. In response to these significant transitions, China is implementing major reforms in health care services with a focus on strengthening primary health care. In this paper, we describe a 12-month diabetes management program, the Happy Life Club™ (HLC™), implemented in a primary health care setting in Beijing, that uses doctor and nurse health coaches trained in behavior change techniques and motivational interviewing (MI). This paper reports the results of this pilot study and discusses issues involved in the implementation of Chronic Diseases Self-Management Programs in China. The intervention group showed improvements in HbA1c levels at 6 months and both the control and intervention groups showed reductions in waist circumference over time. Systolic blood pressure improved over time in the intervention group. The intervention group showed improvement in quality of life across the intervention period and both groups showed decreases in psychological distress across the intervention. Doctor visits increased between baseline and 6 months, but there was no change in doctor visits between 6 and 12 months for both groups. The effects were modest, and further investigations are required to evaluate the long-term impact of health coach approaches in China.

## Introduction

China is now following the trajectory of many Western countries in terms of population aging, increased prevalence of chronic diseases and reductions in the population frequency of healthy lifestyle behaviors. For example, over the period 1998–2008, the incidence of diabetes mellitus (DM) tripled in China and, from 1991 to 2006, physical activity levels decreased by 32% (1, 2). In response to these significant transitions, China is implementing major reforms in health care services for its 1.4 billion citizens. The primary health care reforms, first announced in 2009, aim to deliver basic chronic disease care through community health services with referral of complex cases to the tertiary hospital system (3, 4). Chronic illness management approaches in China are neither typically patient centered nor do they include a central role for the patient in the self-management of their condition (5). Furthermore, patients are often dissatisfied with the medical services they receive while doctors focus on providing medications to manage chronic diseases rather than the facilitation of behavior change to moderate or control these conditions (6, 7).

Our work in China has focused on designing diabetes management programs that can be delivered effectively and efficiently in primary health care settings. Over the last decade the number of cases of DM in China has increased to the extent that China now has the highest number of DM cases globally. Xu et al. (8) estimated that in 2010, the prevalence of DM was 11.6%, representing 113.9 million adults. The prevalence of DM increases with age in Chinese adults: in those aged 70 years and over it is estimated that 21.8% of women and 22% of men have diabetes (9). The Chinese government has recognized the need for new approaches to the management of DM including self-care education and the incorporation of healthy lifestyle interventions into routine care (10, 11). However, such approaches need a trained workforce of health professionals who understand and embrace patient-centered care and who possess the requisite skills in behavior change and counseling principles and practice. Such an approach, involving the training of doctors and nurses in patient-centered care and behavior change techniques, has been piloted and developed into a diabetes self-management program in Beijing, China: the Happy Life Club™ (HLC™). This program is based on a similar program developed in Australia (12).

The HLC™ program involves nurses and doctors in primary care settings, trained in motivational interviewing (MI) and behavior change techniques, delivering face-to-face and telephone coaching to patients with T2DM. MI is a way of communicating with patients that is collaborative in style and focused on how patients talk about change (13). In order to facilitate change, it is assumed that the patient needs to elicit their own ideas about change as they will then be more likely to act. MI is founded upon an attitude of acceptance and compassion. It aims to strengthen the patient’s motivation to achieve a goal by resolving ambivalence. MI is linked to the stages of change approach whereby the coach assists the patient to work through stages of change, from no intention to change to a commitment to change and action (14). The MI approach has been shown to be effective in achieving glycemic control in adults with T2DM but to date the approach has not been tested widely in the Chinese context (15). Therefore, the purpose of this paper is to report findings from the pilot HLC™ study and discuss issues involved in implementing a CDSMP developed for Western primary care in a Chinese setting.

## Materials and Methods

### Sample and program setting

The data reported in this article are based on a 12-month pilot study of *n* = 100 patients of age 55 years and over with Type 2 Diabetes Mellitus (T2DM). The pilot study was conducted in Fangzhuang. The Fangzhuang community is located in the south of Beijing and has a resident population of 110,000; 21.4% of which are aged 60 years and above. The community has an established community health service system that includes a large Community Health Center (CHC) or community hospital which, administrated by the local government, functions as the main primary health care provider. The CHC includes five community health stations (CHSs), which aim to serve the health needs of the local communities. Participants were approached consecutively as they attended their usual diabetes appointment and asked if they wished to participate in the study. Recruitment continued until 100 patients had agreed to participate. The patients were randomly allocated to the intervention group or the control group (see below). Health professionals in the CHC and the CHSs are government employed doctors and nurses. The pilot study was subsequently expanded into a pragmatic cluster randomized controlled trial (16). The study was approved by the Monash University Human Research Ethics Committee.

### The intervention

The HLC program uses trained health coaches. In the pilot study, the control group received usual care provided by a family physician where patients are typically referred to diabetes specialists and/or Traditional Chinese Medicine (TCM) practitioners. The intervention group received telephone and face-to-face coaching in addition to usual care. The key components of the intervention were patient-centered care and the use of MI (13) to help effect change in diet, physical activity, and general chronic disease self-management behaviors. In the first 3 months, participants received two face-to-face and two telephone coaching sessions per month after which, as the participants gained confidence in self-management, the frequency diminished. Overall, the intervention group received a maximum of 19 telephone coaching and 18 face-to-face coaching sessions. The intervention ran for 12 months.

### Coach training

The health coaches (experienced doctors and nurses) received a certified training program. Doctors and nurses were chosen to deliver the intervention as they are by far the main providers of health care in China. Other health professionals such as diabetes educators are virtually non-existent in the Chinese health system (17). The training program consists of a self-learning package and health coach skills workshops. The self-learning package included key concepts in patient-centered care, health psychology and behavior change approaches, the epidemiology of diabetes, and the role of MI in behavior change. The self-learning package was followed by a 2-day intensive MI workshop. This workshop covered the concepts and spirit of MI including: promoting a patient-centered approach and a collaborative coach style that focused on the stage of behavior change of targeted lifestyle behaviors relevant to chronic disease management; eliciting patient’s intrinsic motivation to change; promoting client choice; building self-efficacy; and resolving patient ambivalence. The workshop included the application and practise of MI core skills: the use of open-ended questions; affirmation; reflection, and summarizing across the behavior change process. During the implementation of the HLC™, refresher workshops were conducted and, 1 month after the initial training, the coaches participated in a further half-day advanced training workshop.

### Measures and statistical procedures

Clinical, self-reported health, and well-being measures, and health service use were collected at baseline, 6 and 12 months. Clinical measures included HbA1c, blood pressure, waist circumference, and BMI. Quality of life was measured using the WHOQOL-BREF (18). Psychological well-being was measured using the Kessler Psychological Distress Scale (K10) (19). Participants were asked how often they had visited the doctor in the last 6 months. Differences between the control and intervention groups at baseline, 6, and 12 months were assessed using repeated measures ANOVA with the control and intervention groups as between-subjects factors. Effect sizes were calculated.

## Results

Table 1 shows the baseline characteristics of the participants (*n* = 100). At the 6-month follow-up, *n* = 5 participants were lost to follow-up: one participant died and four participants moved house and could not be contacted. There were no differences between the groups in terms of key demographic variables at baseline except that the control group participants were more highly educated. Sixty-seven percent of the total sample was women.

**Table 1:** **Baseline characteristics of participants**.

Baseline characteristics	Control	Intervention	Total
Participants, *n*	50	50	100
Age in years, mean ± SD	63.3 ± 7.8	65.8 ± 7.5	64.2 ± 7.7
Female	33	34	67
Married (including *de facto*)	47	44	91
Retired	44	46	90
Education			
Primary or less	7	13	20
Secondary/high school	25	34	59
Tertiary/technical	18	3	21
Duration of diabetes in years, mean ± SD	8.2 ± 6.1	9.0 ± 6.3	8.6 ± 6.2

Table 2 shows a comparison between the control and intervention groups at baseline, 6, and 12 months on key clinical and health measures.

**Table 2:** **Mean scores and SD for clinical, self-reported health, and well-being measures and doctor visits at baseline, 6 and 12 months for the control and intervention groups (*n* = 100)**.

Measure	Baseline (*N* = 100)		6 months (*N* = 95)		12 months (*N* = 95)		Effect Size from group (partial eta squared)	Effect Size from time (partial eta squared)
	Control	Intervention	Control	Intervention	Control	Intervention	
HbA1c	7.00 ± 0.81	7.16 ± 1.11	6.96 ± 0.92	6.88 ± 1.10	7.16 ± 1.16	6.88 ± 0.88	0.036 (small effect)	0.029 (small effect)
Systolic blood pressure (mmHg)	128.2 ± 12.7	132.0 ± 15.4	128.6 ± 15.0	129.2 ± 12.1	129.1 ± 12.6	125.0 ± 12.4	0.054 (small effect)	0.026 (small effect)
Diastolic blood pressure (mmHg)	76.5 ± 6.9	77.6 ± 8.1	75.9 ± 6.7	76.6 ± 6.9	76.5 ± 7.8	76.2 ± 7.4	0.008	0.007
Waist circumference (cm)	91.82 ± 7.10	90.56 ± 9.19	89.86 ± 7.25	89.02 ± 8.89	89.13 ± 7.14	88.59 ± 9.26	0.004	0.088 (small-medium effect)
BMI (kg/m2)	25.37 ± 2.64	25.60 ± 3.40	25.47 ± 2.35	25.63 ± 3.27	25.44 ± 2.39	25.60 ± 3.41	0.001	0.001
WHOQOL-BREF	3.40 ± 0.85	3.34 ± 0.67	3.40 ± 0.74	3.44 ± 0.77	3.36 ± 0.49	3.83 ± 0.81	0.047 (small effect)	0.044 (small effect)
K10	19.0 ± 6.4	17.3 ± 6.3	16.9 ± 6.7	15.1 ± 6.2	15.9 ± 5.0	13.8 ± 3.7	0.001	0.159 (medium effect)
Number of community doctor visits	5.38 ± 3.33	5.17 ± 2.49	5.70 ± 4.21	6.88 ± 4.95	5.68 ± 3.87	6.98 ± 4.97	0.016	0.032 (small effect)

*Partial eta squared values: small effects indicated by 0.02, medium effects by 0.13, and large by 0.26*.

There was a significant interaction effect between HbA1c and group over the period baseline to 6 months (*F* = 7.098, *p* = 0.009). The intervention group showed significant improvement in HbA1c levels between baseline and 6 months. However, the effect size was small. Neither group showed changes in HbA1c over the period 6–12 months. There was a significant interaction effect between systolic blood pressure and group (*F* = 5.194, *p* = 0.006), indicating that the intervention group significantly improved over time compared to the control group. Again, the effect size was small. Diastolic blood pressure and BMI did not change across the intervention period for either group; however, waist circumference decreased for both groups over time (*F* = 8.591, *p* < 0.001). There was no significant difference between the groups in terms of decrease in waist circumference. The effect size was small to medium.

There was a significant interaction effect between quality of life and group (*F* = 4.612, *p* = 0.011). The intervention group showed improvement in quality of life across the intervention. The effect size was small. The control group showed no significant change in quality of life across the intervention period. Both groups showed a decrease in scores on the K10 between baseline and 6 months (*F* = 11.306, *p* < 0.001) and between 6 and 12 months (*F* = 4.577, *p* = 0.035), but there were no significant differences between the groups. The effect size for changes over time was medium. In terms of visits to the community doctor in the last 6 months, both groups showed a significant increase in doctor visits between baseline and 6 months (*F* = 4.844, *p* = 0.030), but no change between 6 and 12 months. The effect size for changes over time was small.

## Discussion

The pilot study demonstrated that a CDSMP using Western concepts of behavior change and MI has an effect on the management T2DM particularly in terms of the key physiological parameters of HbA1c levels and systolic blood pressure. However, the effect sizes were small. By 6 months, the intervention group had achieved the goal of an HbA1c <7% and this may have contributed to no further significant reductions in HbA1c at 12 months. The intervention group also showed improvements in quality of life across the intervention and both groups showed reductions in psychological distress.

Both groups showed improvements in some of the clinical and health indicators. This may be due to participation effects, with no differential effect due to the intervention. In China, people with T2DM do not regularly monitor their condition, including HbA1c levels, due to cost. The control group received feedback about their HbA1c levels and this may have motivated them to implement self-management approaches (Hawthorne effect). The study was conducted in a residential area where there was the potential for contamination between the groups. The participants lived in the same building or residential area and potentially had close interaction when shopping or participating in community activities. Participants in the intervention group may have discussed their coaching with other residents. While the coaches were asked not to use MI with their other patients, it is difficult to control this. Coaches may have used the techniques with patients outside the intervention group.

While one of the aims of a CDSMP is to reduce hospital-based specialists’ visits in order to reduce health care costs, in our pilot community, doctor (general practitioner) visits increased. We concluded that this increase was largely due to an improvement in doctor–patient relationships. In China, dissatisfaction with the services provided by hospital-based doctors is very high (20). The HLC pilot may have increased the participant’s confidence in gaining a higher quality of care from their community doctors thus increasing primary health service use.

There are few trials of behavioral and psychological approaches to the management of T2DM in China. One recent but small 12-week intervention (*n* = 40) that used cognitive behavior therapy (CBT), found that the CBT group showed reductions in fasting glucose, HbA1c, and depression compared to the usual care group (21). A systematic review of lifestyle interventions aimed at preventing T2DM in developing countries (22) identified only one Chinese study (23). There is a pressing need to rigorously evaluate the different behavioral and psychological approaches to the management of T2DM in China, particularly when interventions that have only been proven effective in Western settings are used.

We have attempted to address the issues raised by this pilot study in the full pragmatic cluster randomized controlled trial (11) where there is more geographical separation between the groups. The full trial is also sufficiently powered to detect differences between the groups. The pilot study was conducted in a relatively high SES urban area in Beijing. Its applicability in rural and low SES areas is unknown and requires further testing. In the full trial, which includes sampling across SES groups, the influence of SES will be examined.

CDSM approaches require a health workforce that is highly trained in communication skills, patient-centered approaches, and behavior change frameworks and skills. Traditionally, there has been little focus on these skills in health care practitioner training in China (24). Behavior change and psychological approaches to the prevention and management of chronic illnesses are limited in China as there are only 2.4 professionals with psychology training per 1 million of the population (25) compared to 3500 nurses and physicians per 1 million of the population (17). Consequently, psychological and behavioral approaches to health and illness have not been widely endorsed either by medical practitioners, policy makers, or the general population. We were therefore interested in whether a Western model of CDSM would successfully translate into a Chinese setting. It would seem that health literacy concerning chronic illnesses in China is improving, especially among women and those with higher SES (“a long illness turns a patient into a doctor”) (26). In addition, the holistic mind–body approach embodied in TCM, where it is assumed that health is governed by emotion and thoughts, is actually consistent with CDSM approaches (27). Despite the modest effects found in this pilot study, we were pleasantly surprised by how well our CDSMP was adopted by both the coaches and the patients. We could not put it better than one of our coaches:

MI is a powerful tool. Since using it, both me and the patient have opened our mouths, and have more conversations… We work together and start talking about what small steps to take, and what is the easiest way to go… Patients then have their ideas of targets and a plan. I encourage them constantly… It does not work immediately, but several months later, I find they really get success… if one can maintain a new behavior for 3–6 months, the behavior seems to be a stable life habit.

The patients showed similar positive views about their experiences of the intervention:

Before this project, I was quite negative about my disease and for everything I just relied on my doctors. But now, I can manage the disease by myself. If my blood sugar level is high I will try to find the reason by myself first … because I am the person who knows me better. I do not feel the disease is a huge burden to me anymore and that is really good. (Female, 61 years old, duration of T2DM 8 years)

Our qualitative results support the view that patients appear to have benefited from the approach in terms of changing health behaviors and gaining confidence in managing their T2DM. Patients in the intervention group were also able to reach the HbA1c goal of 7% and improve systolic blood pressure. However, we need stronger evidence to conclude that our approach will lead to long-term changes in T2DM management.

## Conflict of Interest Statement

The authors declare that the research was conducted in the absence of any commercial or financial relationships that could be construed as a potential conflict of interest.

This paper is included in the Research Topic, “Evidence-Based Programming for Older Adults.” This Research Topic received partial funding from multiple government and private organizations/agencies; however, the views, findings, and conclusions in these articles are those of the authors and do not necessarily represent the official position of these organizations/agencies. All papers published in the Research Topic received peer review from members of the Frontiers in Public Health (Public Health Education and Promotion section) panel of Review Editors. Because this Research Topic represents work closely associated with a nationwide evidence-based movement in the US, many of the authors and/or Review Editors may have worked together previously in some fashion. Review Editors were purposively selected based on their expertise with evaluation and/or evidence-based programming for older adults. Review Editors were independent of named authors on any given article published in this volume.
